# A Comparison of Two Invagination Techniques for Pancreatojejunostomy after Pancreatoduodenectomy

**DOI:** 10.1155/2015/894292

**Published:** 2015-03-17

**Authors:** Katarzyna Kusnierz, Slawomir Mrowiec, Pawel Lampe

**Affiliations:** Department of Gastrointestinal Surgery, Medical University of Silesia, 14 Medykow Street, 40-752 Katowice, Poland

## Abstract

*Background.* The aim of the study was to compare two invagination techniques for pancreatojejunostomy after pancreatoduodenectomy. *Methods.* For effective prevention of the development of pancreatic leakage, we modified invagination technique that we term the “serous touch.” We analysed the diameter of the main pancreatic duct, the texture of the remnant pancreas, the method of the reconstruction, pancreatic external drainage, anastomotic procedure time, histopathological examination, and postoperative complications. *Results.* Fifty-two patients underwent pancreatoduodenectomy with pancreatojejunostomy using “serous touch” technique (ST group) and 52 classic pancreatojejunostomy (C group). In the ST group one patient (1.9%) was diagnosed as grade B pancreatic fistula, and no patient experienced fistula grade A or C. In the C group 6 patients (11.5%) were diagnosed as fistula grade A, 1 (1.9%) patient as fistula grade B, and 1 (1.9%) patient as fistula grade C. There was a significant statistical difference in incidents of pancreatic fistula (*P* < 0.05) and no statistical difference in other postoperative complications or mortality in comparison group. Anastomosis time was statistically shorter in the ST group. *Conclusions.* “Serous touch” technique appeared to be easy, safe, associated with fewer incidences of pancreatic fistulas, and less time consuming in comparison with classical pancreatojejunostomy.

## 1. Introduction

Pancreatoduodenectomy (PD) is the treatment of choice for most resectable periampullary tumors (malignant and benign disorders of the pancreas and periampullary region). The pancreatic anastomosis is still Achilles' heel of pancreatic surgery since it involves the highest rate of surgical complications among all abdominal anastomoses [1]. The choice of an anastomotic method may be based on the preference of a surgeon or individual characteristics of each patient. From the technical standpoint, an “ideal” pancreaticojejunal anastomosis would meet the following criteria: applicable to all patients, associated with a low rate of pancreatic anastomotic failure-related complications, and easy to teach [2]. More than 80 different methods of pancreaticoenteric reconstruction have been proposed, illustrating the complexity of surgical techniques as well as the absence of the gold standard [1]. Many factors associated with an increased incidence of its complication have been identified. Among them, a small pancreatic ductal size with a soft pancreas creates one of the technical hurdles to the completion of the anastomosis and is known to be a risk factor for major leakage. Some retrospective or prospective studies have suggested the need for technical modifications to reduce the pancreatic fistula rate [3]. The incidence of PF is estimated to be 5% to 30%, which varies according to the definition [4].

For effective prevention of the development of the pancreatic leakage, we modified the invagination technique that we term the “serous touch” technique. Our technique is based on the assumption that wounds causing tissue adhesion after surgical operation appear to be related to the serous membrane covering viscera and the serosa plays a role in the healing of the damaged organs [5].

According to the assumptions the anastomosis takes the advantage of the properties of the serosal membrane (fast healing) and its adherence to the pancreas, as it facilitates the healing. Additionally, a cuff made of the intestine, which adheres closely to the pancreas, should ensure the tightness of the anastomosis. Hypothetically, all the factors mentioned should result in a smaller number of PFs, as compared to classical invagination technique in which the mucous membrane adheres to the pancreas.

## 2. Materials and Methods

**Figure 1:**
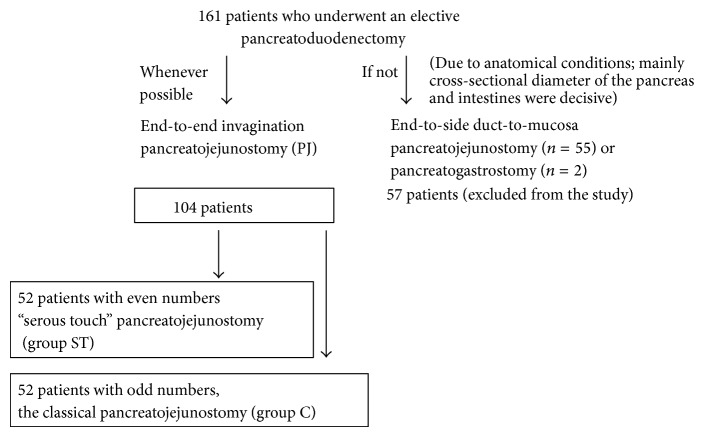
Study eligibility criteria.

Between January 2009 and December 2011 patients who underwent an elective pancreatoduodenectomy in our Department of Gastrointestinal Surgery were divided into two groups (Figure 1). We performed end-to-end invagination pancreatojejunostomy (PJ) whenever possible. Anatomical conditions, mainly cross-sectional diameter of the pancreas and intestines, were decisive. In the remaining cases, when the conditions did not allow for the implementation of end-to-end anastomoses, end-to-side duct-to-mucosa pancreatojejunostomy or pancreatogastrostomy were performed instead. These anastomoses were excluded from the study. Among the cases eligible for the end-to-end invagination technique, we created two groups that we analyzed: patients who underwent PD with PJ using our modified invagination technique that we term the “serous touch” (group ST) and the classical pancreatojejunostomy (group C). Qualification to the groups of classical anastomosis or “serous touch” took place independently of the operating surgeon, alternatively (1 classical anastomosis, 1 serous touch).

In both groups we analyzed intraoperative factors: the diameter of the main pancreatic duct, the texture of the remnant pancreas, the method of the reconstruction, pancreatic external drainage, estimated blood loss, total operative, and anastomotic procedure times, as well as histopathological examination and postoperative complications. A statistical analysis was performed to check if the soft remnant or the external drainage of the pancreatic duct influences PF.

### 2.1. Operative Procedure

**Figure 2:**
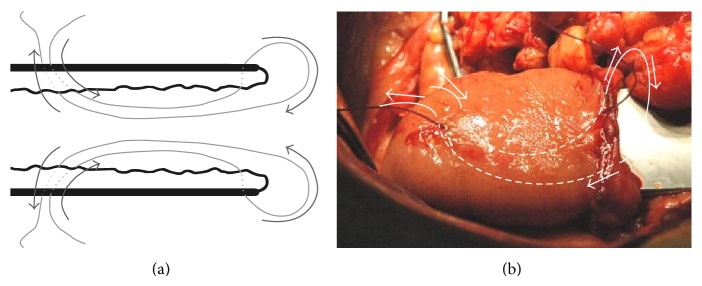
((a), (b)) The technique of placing 1 of 3 sutures allowing the creation of the intestinal cuff.

**Figure 3:**
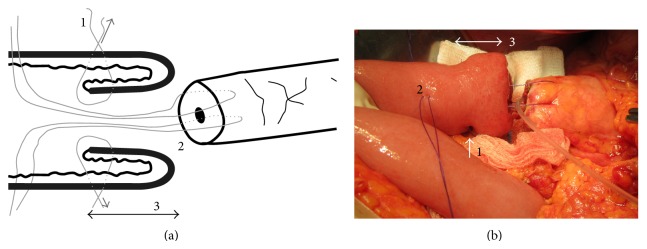
(a) A diagram of pancreaticojejunostomy modification. (1) Sutures which fix the intussusception of the intestinal wall. (2) Sutures which allow drawing the cross-section of the pancreas into the cuff made of intestine. (3) A cuff made by the intussusception of the intestinal wall. (b) Cut end of the pancreas with sutures put through the entire thickness. The pancreas is drawn into the bowel by means of these sutures.

**Figure 4:**
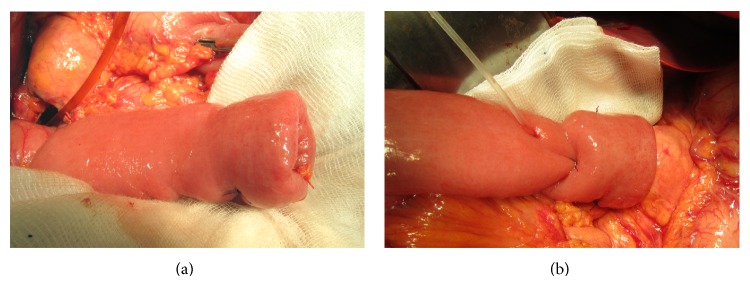
(a) Intestinal cuff into which the cut end of the pancreatic remnant is drawn. Visible suture fixing intussusception of the intestine wall. (b) The pancreaticojejunostomy and the drain from Wirsung's duct.

Four surgeons performed the anastomoses; however, one of the authors, Pawel Lampe, supervised all the operations. Our modified technique of end-to-end PJ is shown in Figures 1–3. The pancreas is transected with an electrocautery on the scheduled line. Afterwards a hemostasis is performed. The main pancreatic duct is identified. The cut end of the pancreatic remnant is mobilized for approximately 2.5–3 cm to allow its intussuscepting into the intestine. We start with the intestine preparation for the anastomosis. We insert the first out of the three sutures, which will create the intestinal cuff into which the pancreas is intususcepted (3-0 synthetic absorbable monofilament suture) (Figures 2 and 3). These three sutures are put 5-6 cm from the edge of the intestine, so that the cuff is 2.5–3 cm (Figures 2 and 3). After putting three sutures and tying the knots we get the intestine intussusception, cuff (Figures 3 and 4). If we assume that the mesentery connects to the intestine at the 6 o'clock position, we put the sutures at 8 o'clock, 12 o'clock (the antimesenteric side), and 4 o'clock positions. Depending on the diameter of Wirsung's duct and the texture of the pancreas we insert a drain into Wirsung's duct and fix it with 5-0 absorbable sutures to the duct (Figures 3 and 4). The drain is used for external drainage of the pancreatic duct. The drain is fixed to the jejunal wall with Witzel's method using 4-0 synthetic absorbable monofilament suture. The jejunal limb is moved to the pancreatic cut end by a retromesenteric route. Then we begin the pancreatic anastomosis with the intestine with two sutures put on the intestine at around 3 o'clock and 9 o'clock positions (synthetic long-term absorbable monofilament suture, UPS metric size 0 or 1). We put on a suture 4–4.5 cm from the cuff's edge from the outer surface to the inner surface throughout the entire thickness of the bowel, and then the same suture is put on through the thickness of the pancreatic remnant (one suture at both sides of Wirsung's duct) (Figure 3). Next we return again through the full thickness of the bowel. We put on 2 sutures of the type by means of which we draw the pancreas into the formed cuff (Figure 3). After the intussusception of the pancreas into the cuff, the sutures are tied (Figure 4). Next we put on a few additional single sutures (6-8 sutures) connecting the pancreas and the seromuscular layer of the jejunum (4-0 synthetic absorbable monofilament suture).

In the C group the end-to-end invagination pancreatojejunostomy was performed with two single suture layers (4-0 synthetic absorbable monofilament suture). The first layer connected the pancreatic parenchyma with the full thickness of the jejunum, and the second connected pancreatic parenchyma with jejunal seromuscular layer.

All patients had one drain placed in close proximity to the pancreatic anastomosis during the operation. In both groups the pancreatic drain was utilized in the case of the soft pancreas and if the pancreatic duct diameter was ≤3 mm.

*Complications Definitions.* Pancreatic fistulas were defined by measurable pancreatic fluid output after postoperative day 3 (containing more than three times the normal serum amylase level) with clinical signs of an infection and/or necessitating a change in the clinical management. According to the ISGPF definition, the outcomes were divided into the following grades: grade A: biochemical fistula without clinical consequence; grade B: fistula that shows clinical symptoms or requires any therapeutic intervention; grade C: fistula with severe clinical consequence. Fluid collection (abscess) definition is as follows: fluid collection at least 5 cm in diameter diagnosed with ultrasound or CT associated with presence of pus on guided aspiration carried out for clinical fever with leukocytosis/leucopenia (patients in septicaemia), tachycardia, and local abdominal tenderness with or without prior evidence of acute pancreatitis and following removal of drains [6]. DGE was defined by the need for maintenance of the nasogastric tube (NGT) for 3 days, need for reinsertion of NGT for persistent vomiting after postoperative day 3, or inability to tolerate a solid diet by postoperative day 7 [7]. Postoperative pulmonary complications were defined as pneumonia with evidence by radiologic pulmonary infiltrates and/or the presence of pathogenic bacteria in the sputum culture, and pulmonary atelectasis required frequent bronchoscopic toilet or prolonged ventilator support [8]. Postoperative pulmonary, cardiac, and neurological complications were defined as any postoperative adverse event meeting Classification of Surgical Complication Adopted for Pancreatic Surgery criteria for a grade II or higher [9].

All reviewed procedures were conducted according to the principles outlined in the Declaration of Helsinki.

The results of the quantitative data analysis are expressed as mean ± standard deviation (SD), indicating the minimum and maximum values. The results of the qualitative data analysis are presented as percentages. In the case of the quantitative data normality was checked with the Shapiro-Wilk test. The following tests were used: in the case of normal distribution the Student *t* parametric test was used, and in the case of nonnormal distribution, nonparametric Mann-Whitney *U* test was used. In the case of the qualitative data nonparametric tests were used depending on the size of the group: Chi-square, Yates corrected Chi-square, and *V*-square test. As the statistically significant result was taken the *P* value *P* < 0.05. All analyses were performed with the statistical software Statistica 10.0 (StatSoft, Inc.).

## 3. Results

Between 1 January 2009 and 8 December 2011, 161 patients underwent an elective pancreatoduodenectomy in our Department of Gastrointestinal Surgery. A hundred and four patients underwent end-to-end invagination anastomoses (Figure 1). Fifty-two patients underwent PD with PJ using our modified “serous touch” technique (group ST) and 52 with classical pancreatojejunostomy (group C). Fifty-seven patients underwent other than end-to-end invagination technique anastomoses and were excluded from the study.

*Patients Characteristics and Analyzed Factors.* Among the 52 patients in the ST group, 35 (67.3%) underwent surgery because of diagnosed malignant tumors, 17 due to benign tumors, in the C group 37 (71.2%) and 15, respectively. There was no statistical difference in patients' characteristics between the two groups (Table 1). In the patients' history we found the following cardiovascular diseases: coronary artery disease, hypertension, mitral valve prolapse, cardiomyopathy, and arrhythmia. Among pulmonary diseases we had emphysema, chronic bronchitis, and chronic obstructive pulmonary disease. The postoperative drain duration was 3 days. In 1 case (1.9%) in the ST group and in 8 cases (15.4%) in the C group the drain duration was 7 days because of elevated 3x normal amylase level in the drain. The PJ was stented (external drainage) in 30 cases (57.7%) in the ST group and in 23 (44.2%) in the C group. The stent duration was 21 days. Anastomosis time, one of the primary endpoints of this study, was statistically shorter in the ST group than in the C group (*P* < 0.0001). The differences in intraoperative factors and histopathological examination are shown in Table 2.

In the ST group one patient (1.9%) was diagnosed as grade B PF and required a conservative treatment. In the C group 6 patients (11.5%) were diagnosed as PF grade A, 1 (1.9%) patient as fistula grade B, and 1 (1.9%) patient as fistula grade C. A statistically meaningful difference was found in PF between the two groups. Considering PF B and C only, there was no statistical difference. Carrying out a statistical analysis of the dependency between the number of soft pancreas cases in the ST and C groups and the number of pancreatic fistulas, there was no statistically significant difference between the two groups; soft pancreas diagnosis did not affect the incidence of fistulas (Yates corrected Chi-square test, *P* ≥ 0.05). Carrying out a statistical analysis of the dependency between the use of pancreatic stent in the ST and C groups and the presence of pancreatic fistulas, there was no statistically significant difference between the two groups; the application of the stent did not influence the incidence of fistulas (Yates corrected Chi-square test, *P* ≥ 0.05).

One (1.9%) patient in the ST group developed complication such as intraperitoneal bleeding from the remnant part of the uncinate process and required reoperation 6 hours after the pancreatoduodenectomy and one (1.9%) patient with abdominal infection (abscess) required percutaneous drainage (interventional radiology). In the C group three (5.8%) patients with abdominal fluid collections (1 abscess) required drainage (interventional radiology) and 1 (1.9%) eventration (required reoperation). Other complications were cured conservatively (nutritional support, antibiotic coverage). Pulmonary complications included pneumonia in 3 patients from the ST group and in 3 from the C group; cardiac complications included 1 arrhythmia and 1 myocardial ischaemia in the ST group and 1 arrhythmia in the C group. In group C there was a case of 1 neurological complication: transient ischaemic attack. There was no statistical difference in postoperative complications and mortality in ST and C groups (Table 3).

## 4. Discussion

Modifying our method we used the healing properties of the serosa and assumed that the cuff made from the intestine will ensure good adhesion of the serosa to the surface of the pancreas, which will improve the tightness of the anastomosis. We assumed also that a smaller number of stitches put between the pancreas and the intestine will reduce trauma to the pancreas, as well as shortening the time of the anastomosis. Analyzing our results, we found that although the time of the “serous touch" anastomosis was significantly shorter comparing with classical anastomosis, a few minutes are not significant taking into account the duration of the whole operation. However, reduction of the number of PFs, which was the main aim of the anastomosis modification, was achieved.

There are many methods and technical details of the pancreatic-intestinal anastomosis, the aim of which is to reduce the risk of pancreatic fistula and, thus, postoperative mortality. To make the test results and the effectiveness of the method comparable, standardization of the definition of the PF and its severity is necessary. Most current and useful definition and grading of PFs by severity is created by the International Study Group on Pancreatic Fistulas (ISGPF) [10, 11]. In pancreatic surgery, grade A PF is acceptable; grades B and C are crucial.

Anastomosis between the pancreatic end and the jejunum is performed as either end-to-side duct-to-mucosa anastomosis, end-to-side (dunking), or end-to-end invagination anastomosis [11, 12]. It is difficult to speak of the superiority of the invagination technique over others, because the selection and use of an appropriate method depend on many factors. One of them is the ratio of the diameter of the pancreas to the diameter of the lumen of the intestinal loop, which sometimes prevent the performance of the end-to-end anastomosis. Some authors prove that the invagination technique was safer in high-risk patients with small ducts or soft friable pancreas [11–13]. Yang et al. have a similar view. They recommend their own modified method (modified child pancreaticojejunostomy), in which the end-to-end pancreaticojejunal anastomosis is made with a two-layer polypropylene continuous running suture especially for the operation in a deep position and/or with a soft pancreas [3]. In their material (31 patients) they diagnosed no postoperative pancreatic fistulas; the average operative time (pancreaticojejunostomy) was 14.2 minutes. We had only 18 patients with soft pancreas in both groups and we proved that soft pancreas did not influence the number of PFs. The time of our anastomosis was comparable.

An interesting modification of the end-to-end anastomosis was presented by Chinese authors as the end-to-end invaginated pancreaticojejunostomy with transpancreatic U-sutures [14]. In their material (88 patients) they found out 2.2% of postoperative pancreatic fistulas. We used similar sutures through the thickness of the pancreas.

A prospective randomized trial published by Peng et al. showed that an absorbable ligature looped around the jejunum, with the invaginated pancreas inside, reduces the number of postoperative PFs [15]. No patient in the 106 patients randomized to the binding group developed leakage, postoperative complications developed in 24.5%; 3 patients (2.8%) died in the perioperative period. Maggiori et al. disagree with arguing that median delay for healing of postoperative pancreatic fistula was longer in the binding pancreaticojejunostomy group and postpancreatectomy hemorrhage was more frequent in the binding PJ [16]. The binding anastomosis could be performed easily, but the tightness of the binding wrap was difficult to control [15]. If the tying is too tight, the blood supply of the anastomosis may be occluded; if it is too loose, pancreatic fluid may leak from the gap between the pancreatic stump and the jejunum [15]. The use of “serous touch” technique allowed us to achieve tight anastomosis without the blood supply disturbances. This anastomosis takes the advantage of the properties of the serosal membrane (fast healing) and its adherence to the pancreas facilitates the healing. In the experimental work Bai et al. compared the three types of anastomoses: end-to-end pancreaticojejunostomy invagination (EEPJ), end-to-side duct-to-mucosa sutured anastomosis (ESPJ), and binding pancreaticojejunostomy (BPJ) [17]. They were assessing the patency of pancreaticoenterostomy and pancreatic exocrine function after the three surgical methods (experimental study). Anastomotic patency was assessed after 8 weeks by body weight gain, intrapancreatic ductal pressure, pancreatic exocrine function secretin test, pancreatography, and macroscopic and histologic features of the anastomotic site [17]. They showed that the biggest intensification of variable degree of occlusion, dilation, and meandering of the main pancreatic duct and cicatricial fibrous tissue within intussusception appeared after EEPJ [17]. Our method of intussusception without putting two layers of sutures allows a good, unforced, and tension-free adhesion of the intestine wall to the pancreas. We based our method on putting a minimum number of sutures between the pancreas and the intestine. A large number of sutures may damage the pancreatic parenchyma (which is important especially in soft pancreas) and can cause scarring in the line of anastomosis with parenchymal ischaemia (especially in two-layer anastomoses) and parenchymal fibrosis. We put two sutures through the full thickness of the pancreas connecting the pancreas with the intestine. Similar sutures in anastomoses between pancreas and intestine or stomach were also used by other authors [2, 18–20].

In an interesting study, Wang et al. used a modified method to incompletely invaginate the pancreatic stump into the jejunal lumen with transpancreatic interlocking mattress sutures [21]. In this study only two patients (2.53%) with grade A and B pancreatic fistula were found and the median time to perform the end-to-end pancreaticojejunostomy was 15.3 min (range 9–24 min).

An important aspect of pancreatic-intestinal anastomosis after PD is the number of layers of the anastomosis. There are supporters of just one-layer anastomosis, who claim that, in case of insufficiency of the first layer, the second does not protect the anastomosis and it is better to have one, well-made layer [22]. However, Ibrahim et al. describe and highlight the advantages of a triple-layer end-to-side duct-to-mucosa pancreaticojejunostomy (1.96% postoperative pancreatic fistula) [23]. There are few works comparing the two PJ methods for approximating the pancreatic parenchyma to the jejunal seromuscular layer: interrupted versus continuous sutures [24]. While in the work of Lee et al. there was no significant difference between the interrupted suture and continuous suture methods for preventing pancreatic fistula, authors discuss the advantages of the continuous suture [24]. Pancreatic fistula occurred in 14 patients (11%) among the interrupted suture cases and in 10 (6%) among the continuous suture cases (*P* = 0.102) [24]. Our “serous touch” technique is a kind of one-layer anastomosis.

Controversies also accompany stenting of the pancreatic-intestinal anastomosis. The problem of stenting the anastomosis, whether to stent and whether to use internal or external drainage, is still unresolved. Some of the works find that neither external nor internal drainage reduces the amount of postoperative PFs [25–27]. In our method, we apply stenting individually, depending on the size of Wirsung's duct and texture of the pancreas.

It is positive that many surgeons attempt to modify the PJ in order to reduce postoperative complications, mainly pancreatic fistulas and postoperative mortality. It is difficult, however, without prospective randomized trials, to determine safety of each type of anastomosis and its modifications. If the anastomoses performed, independently of the technique, are burdened with a small amount of complications, they should be considered safe. At present, the only reproducible factor that is able to significantly reduce morbidity and mortality in pancreaticoduodenectomy is the establishment of high-volume regional centers [28]. Currently, at high-volume centers, the rates of perioperative mortality and morbidity after pancreatoduodenectomy are typically reported at 1%–3% and 30%–40%, respectively [29].

Compared with traditional end-to-end invaginated anastomosis, “serous touch” technique bears the following advantages: (1) simplicity, as only two transpancreatic sutures have to be placed across the pancreatic stump and the jejunum walls, respectively; (2) small amount of sutures traumatizing the pancreas, crucial in soft pancreas; (3) safety, as the intestine cuff closes any gaps between the jejunum and the pancreas remnant; (4) good healing by close adhesion of the intestine serosa to the pancreas.

The limitations of this study include small sample size, anastomoses were performed when the sizes of pancreas and intestine were appropriate and matched each other, only the early results of the performed anastomoses are known, the decision whether to use pancreatic drainage may be at surgeon's discretion and be subjective (soft pancreas), and only “serous touch” technique and classic end-to-end pancreatojejunostomy were compared.

In conclusion, “serous touch” technique appeared to be easy and safe, associated with fewer incidences of pancreatic fistulas in comparison with classic pancreatojejunostomy.

## Figures and Tables

**Table 1:** Patients' characteristics.

Clinical data	ST group (*n*—52)	C group (*n*—52)	*P* value
Age (years) mean ± SD	58.0 ± 13.7	59.3 ± 8.9	*P* = 0.7646^±^
Range	22–79	39–75	
Sex (number male/female)	30/22	27/25	*P* = 0.5545^¶^
Abdominal pain	32 (61.5%)	40 (77%)	*P* = 0.0892^¶^
Loss of body weight	38 (73%)	41 (78.8%)	*P* = 0.4912^¶^
Preoperative biliary drainage	12 (23.1%)	8 (15.4%)	*P* = 0.3220^#^
Jaundice	24 (46.2%)	28 (53.8%)	*P* = 0.4328^¶^
Diabetes mellitus	22 (42.3%)	24 (46.2%)	*P* = 0.6929^¶^
Cardiovascular disease^+^	18 (34.6)	16 (30.8%)	*P* = 0.6759^¶^
Pulmonary disease^+^	4 (7.7%)	1 (1.9%)	*P* = 0.3593^*^
ASA class on admission			
(I) Healthy	7 (13.5%)	11 (21.2%)	*P* = 0.3022^#^
(II) Mild systemic disease	30 (57.7%)	26 (50%)	*P* = 0.4314^¶^
(III) Severe systemic disease	15 (28.8%)	11 (21.2%)	*P* = 0.3650^¶^
(IV) Severe systemic disease that is a constant threat to life	0	1 (1.9%)	*P* = 1.0000^*^

SD: standard deviation; ASA: American Society of Anesthesiologists; ^+^diseases are classified using the Ninth Revision of the World Health Organisation's International Classification of Disease; Yates corrected Chi-square test^*^; Chi-square test^¶^; *V*-square test^#^; Mann-Whitney *U* test^±^.

**Table 2:** Intraoperative factors, tumor characteristic, and histopathological examination.

Clinical data	ST group (*n*—52)	C group (*n*—52)	*P* value
Method of reconstruction			
PPPD	4 (7.7%)	8 (15.4%)	*P* = 0.3572^*^
Whipple	48 (92.3%)	44 (84.6%)	
Diameter of main pancreatic duct (mm): mean ± SD	Range 1–7	Range 1–9	*P* = 0.9119^±^
	2.86 ± 1.27	2.98 ± 1.53	
The soft texture of the remnant pancreas	7 (13.5%)	11 (21.2%)	*P* = 0.3022^#^
Pancreatic external drainage	30 (57.7%)	23 (44.2%)	*P* = 0.1697^¶^
Anastomotic procedure time, mean ± SD (min)	14.48 ± 1.95	16.88 ± 2.08	*P* = 0.0001^±^
	Range 12–20	Range 13–25	
Total operative time, mean ± SD (min)	329.23 ± 54.02	338.75 ± 45.10	*P* = 0.2809^•^
	Range 205–480	Range 240–450	
Estimated blood loss, mean ± SD (mL)	514.13 ± 150.25	560.38 ± 318.45	*P* = 0.7973^±^
	Range 300–1050	Range 300–2500	
Histopathological examination			
Adenocarcinoma	30 (57.7%)	34 (65.4%)	*P* = 0.4201^¶^
Intraductal papillary-mucinous carcinoma	0	1 (1.9%)	*P* = 1.0000^*^
Intraductal papillary-mucinous neoplasm	2 (3.8%)	4 (7.7%)	*P* = 0.6741^*^
Solid pseudopapillary neoplasm	1 (1.9%)	2 (3.8%)	*P* = 1.0000^*^
Neuroendocrine tumor	0	1 (1.9%)	*P* = 1.0000^*^
Neuroendocrine carcinoma	4 (7.7%)	2 (3.8%)	*P* = 0.6741^*^
Tubular adenoma	1 (1.9%)	0	*P* = 1.0000^*^
Serous cystadenoma	2 (3.8%)	0	*P* = 1.0000^*^
Serous microcystic adenoma	1 (1.9%)	0	*P* = 1.0000^*^
Chronic pancreatitis	11 (21.2%)	8 (15.4%)	*P* = 0.4487^#^
Metastatic melanoma	1 (1.9%)	0	*P* = 1.0000^*^

PPPD: pylorus-preserving pancreaticoduodenectomy; SD: standard deviation; Yates corrected Chi-square test^*^, Chi-square test^¶^; *V*-square test^#^; Mann-Whitney *U* test^±^; Student*t*-test^•^.

**Table 3:** Postoperative complications.

Postoperative complications	ST group (*n*—52)	C group (*n*—52)	*P* value^*^
Pancreatic fistula	1 (1.9%)	8 (15.4%)	*P* = 0.0364
Intraperitoneal bleeding (required reoperation)	1 (1.9%)	0	*P* = 1.0000
Acute postoperative pancreatitis	1 (1.9%)	0	*P* = 1.0000
Bile leakage	1 (1.9%)	2 (3.8%)	*P* = 1.0000
Abdominal fluid collections	1 (1.9%)	3 (5.8%)	*P* = 0.6101
Wound infection	4 (7.7%)	3 (5.8%)	*P* = 1.0000
Delayed gastric emptying	6 (11.5%)	4 (7.7%)	*P* = 0.7394
Pulmonary complications	3 (5.8%)	3 (5.8%)	*P* = 0.6741
Cardiac complications	2 (3.8%)	1 (1.9%)	*P* = 1.0000
Neurological complications	0	1 (1.9%)	*P* = 1.0000
Eventration (required reoperation)	0	1 (1.9%)	*P* = 1.0000
Overall morbidity	23 (44.2%)	22 (42.3%)	*P* = 1.0000
Mortality	1 (1.9%)	2 (3.8%)	*P* = 1.0000

^*^Yates corrected Chi-square test.
